# Dr. Charles F. Dinger

**Published:** 1889-05

**Authors:** 


### Obituary.

Dr. Charles F. Dinger died in Baltimore, March 29, 1889, of pneumonia, after an illness of several weeks' duration, in the twenty-seventh year of his age.

Dr. Dinger was born in New York City, May 22, 1862, and was the son of Rev. F. W. Dinger, Pastor of North Avenue Methodist Episcopal Church, of Rochester, N. Y.

Dr. Dinger graduated at the Baltimore College of Dental Surgery in 1882, and later in medicine at the University of Maryland.

Shortly after graduating in dentistry he was appointed a Demonstrator of Operative Dentistry in the University of Maryland Dental Department, which position he held until his death.

He was also Secretary of the Maryland State Dental Association, and Corresponding Secretary of the Alumni Association of the University of Maryland Dental Department, having been elected to the latter position March 13th, 1889, some two weeks before his death.

Dr. Dinger was one of the most promising young men who have of late years entered upon the practice of dentistry, being energetic, and skillful and prominent among the dentists of Baltimore in every enterprise, having for its object the advancement of his profession.

A long and useful life was apparently before him, and his sudden death was a surprise as well as a regret to all who knew him.

